# Supplementary figures and images for: Trypanosomatid selenophosphate synthetase structure, function and interaction with selenocysteine lyase
Source: PLoS Negl Trop Dis. 2020 Oct 5;14(10):e0008091. doi: 10.1371/journal.pntd.0008091 (PMC7595633; doi:10.1371/journal.pntd.0008091)

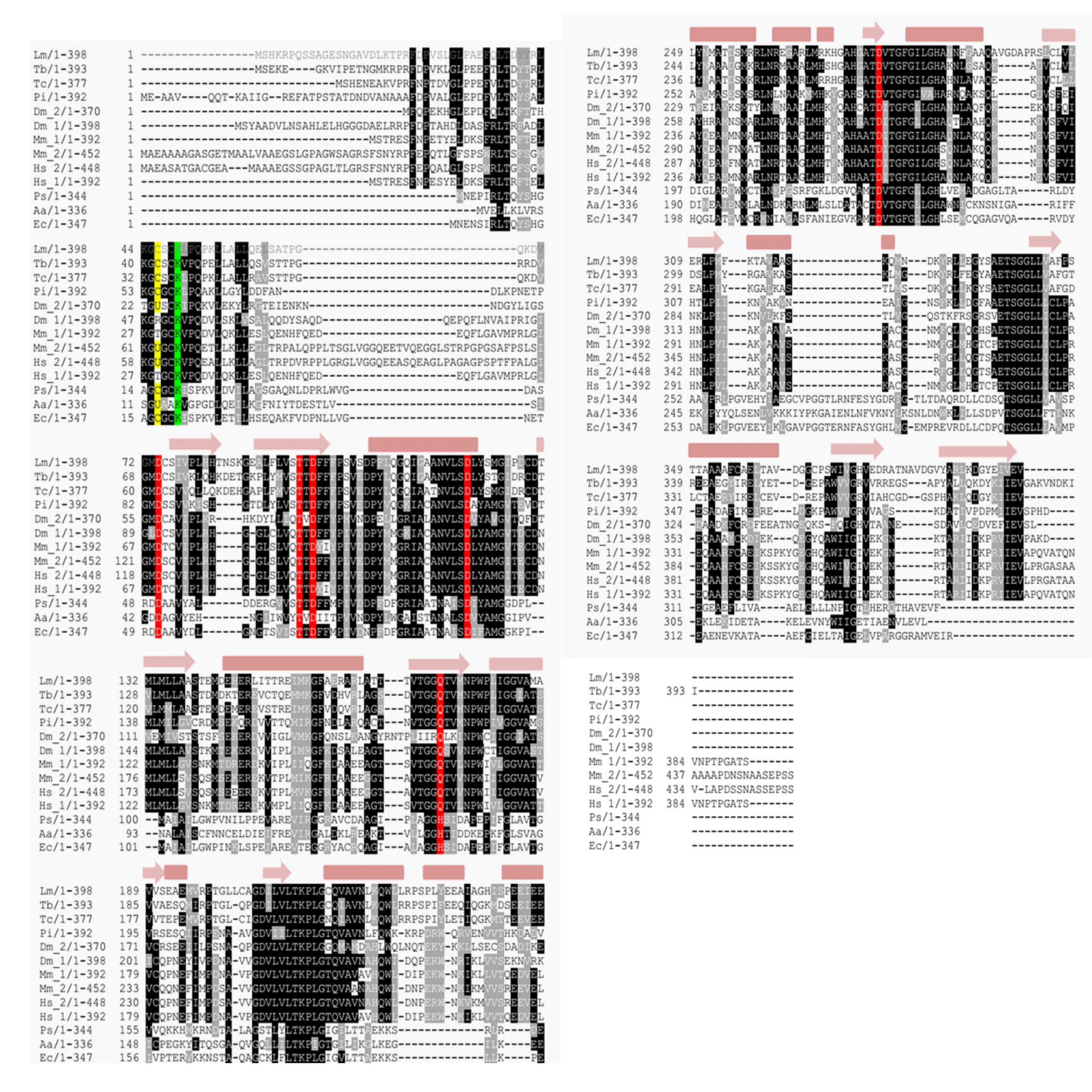

Supplement: S1 Fig — Conserved residues are highlighted (catalytic Cys/Sec in yellow and Lys in green, and ATP binding amino acids in red). Secondary structures are also shown (arrow: α-helix; rectangle: β-strand). Amino acid sequences: Lm–L. major (XP_001687128.1), Tb–T. brucei (EAN78336.1), Tc–T. cruzi (PBJ75389.1), Pi–Phytophthora infestans (EEY58478.1), Dm—Drosophila melanogaster (Dm_1: AAB88790.1, Dm_2: NP_477478.4), Mm—Mus musculus (Mm_1: AAH66037.1, Mm_2: AAC53024.2), Hs–Homo sapiens (Hs_1: AAH00941.1, Hs_2: AAC50958.2), Ps—Pseudomonas savastanoi (EFW86617.1), Aa–Aquifex aeolicus (WP_010880640.1), Ec—Escherichia coli (KPO98227.1). (TIF) [file pntd.0008091.s001.tif]

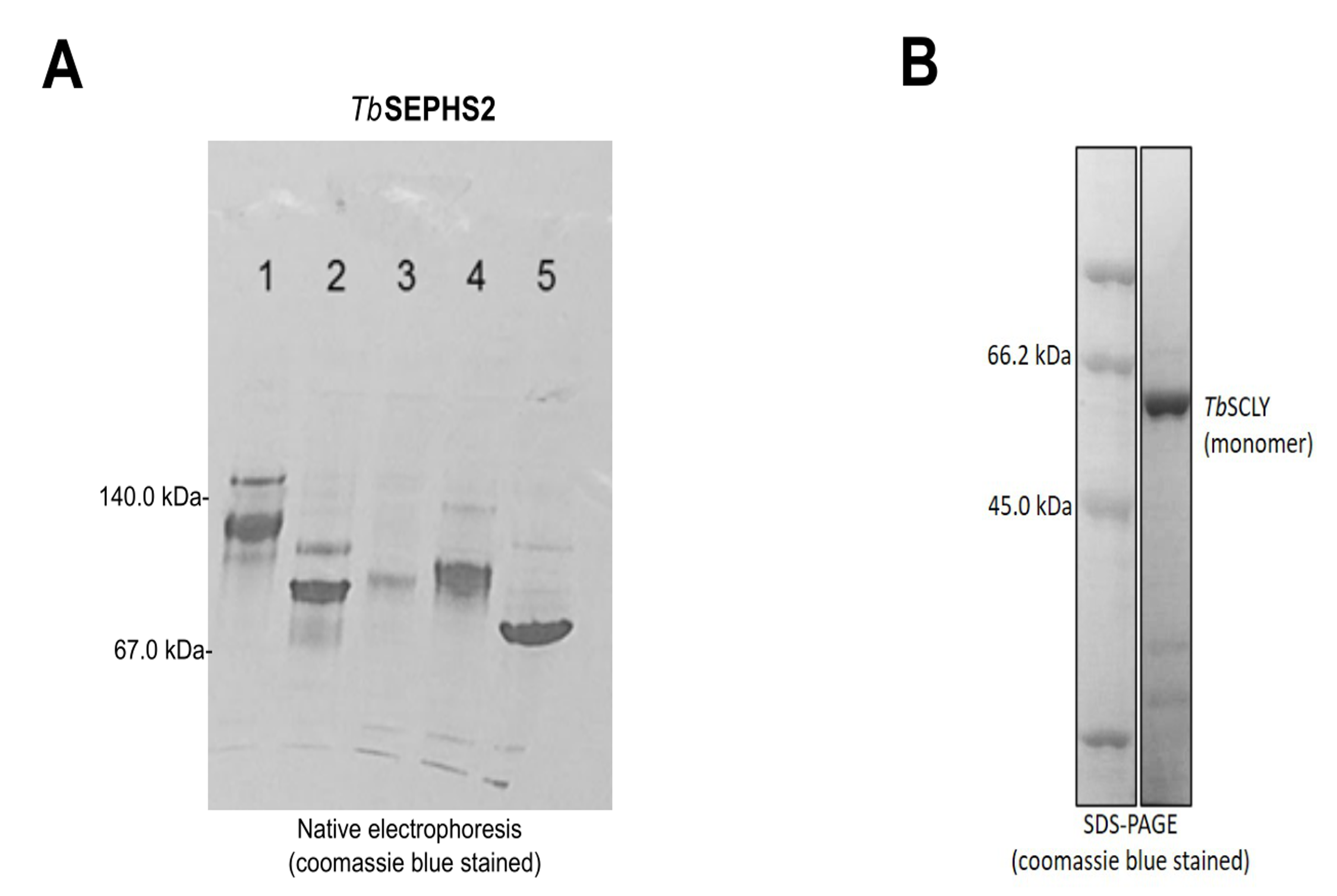

Supplement: S2 Fig — A- Coomassie blue-stained native gel electrophoresis of T. brucei and L. major selenophosphate synthetase constructs: 1- TbSEPHS2, 2- ΔN(70)-TbSEPHS2, 3- ΔN(25)-TbSEPHS2, 4- LmSEPHS2, and 5- ΔN(69)-LmSEPHS2. Major bands correspond to dimers. The second most abundant species in each lane corresponds to the respective tetramer. B- Coomassie blue-stained SDS-PAGE of T. brucei selenocysteine lyase. (TIF) [file pntd.0008091.s002.tif]

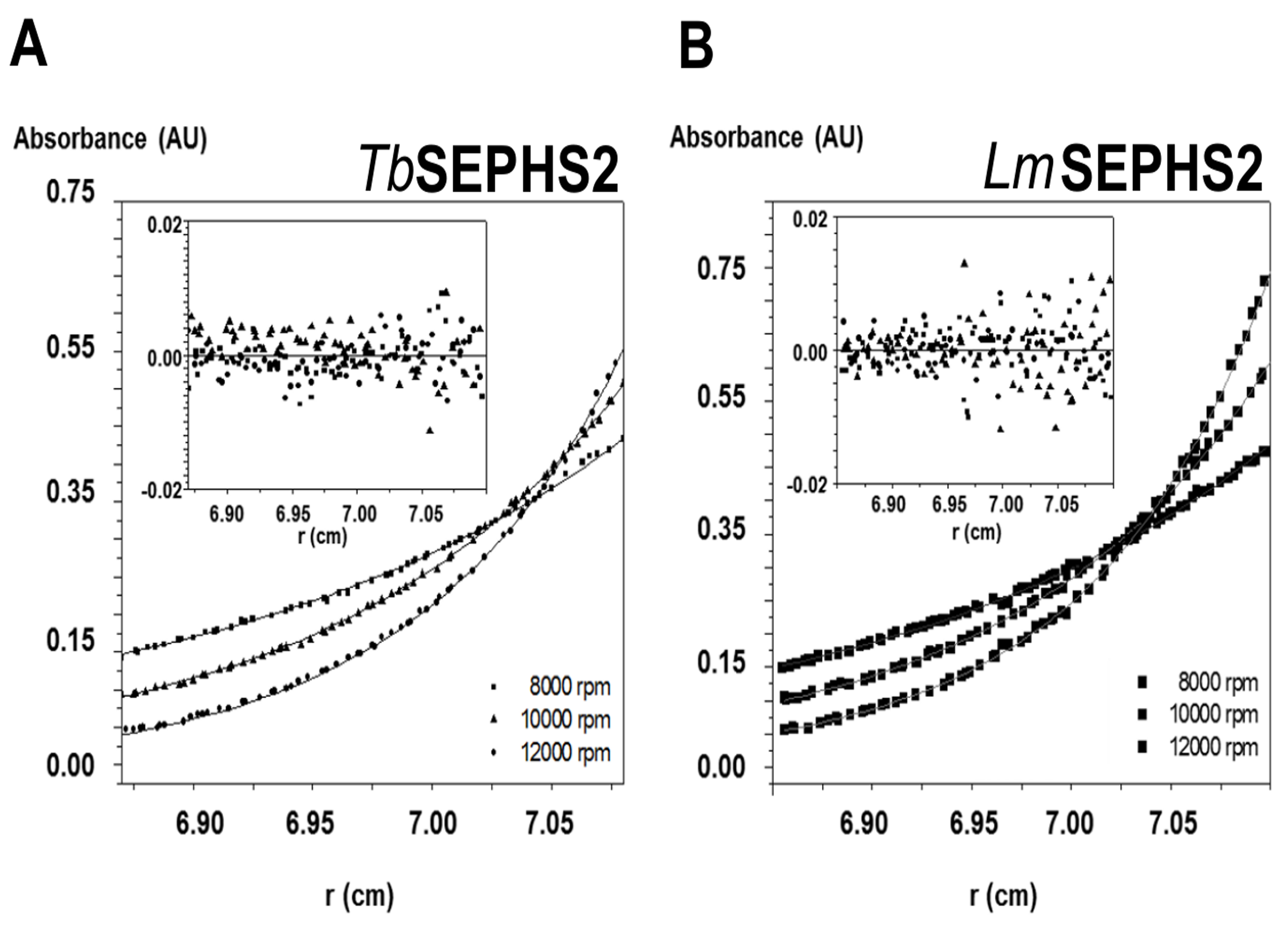

Supplement: S3 Fig — Multi-speed and multi-concentration global fitting for a dimer-tetramer self-association system of A- TbSEPHS2 (Kd = 161 ± 10 μM) and B- LmSEPHS2 (Kd = 178 ± 10 μM). Fitting residuals are shown as insets. (TIF) [file pntd.0008091.s003.tif]

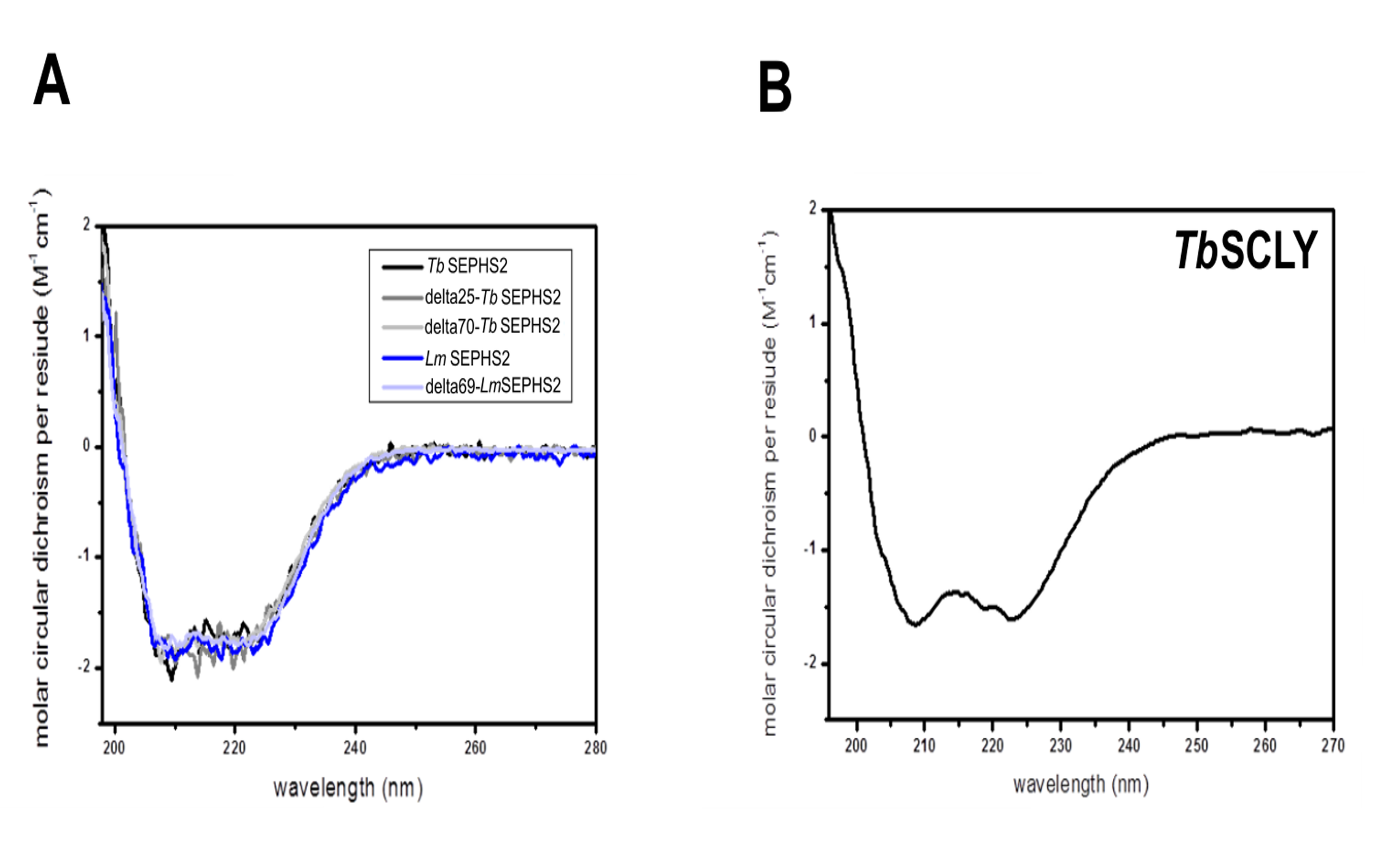

Supplement: S4 Fig — CD spectra for A- selenophosphate synthetase constructs (TbSEPHS2, ΔN(25)-TbSEPHS2, ΔN(70)-TbSEPHS2, LmSEPHS2 and ΔN-LmSEPHS2, and B- T. brucei selenocysteine lyase (TbSCLY). (TIF) [file pntd.0008091.s004.tif]

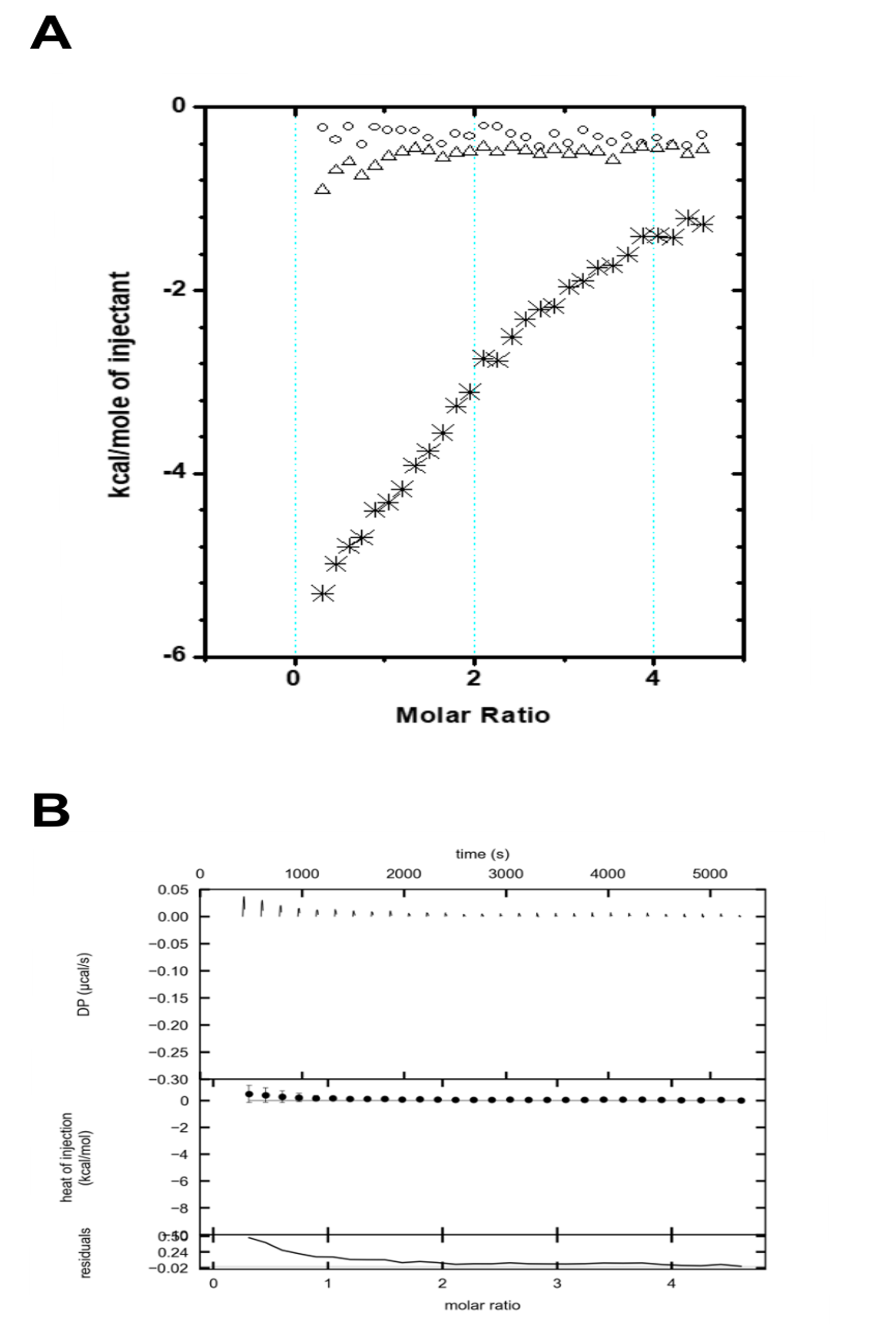

Supplement: S5 Fig — A- ITC data for SCLY-buffer (circle), buffer-SEPHS2 (triangle) and SEPHS2-SCLY (star), and B- SCLY-ΔN(70)-SEPHS2 titration experiments using VP-ITC calorimeter and analyzed in NITPIC. (TIF) [file pntd.0008091.s005.tif]

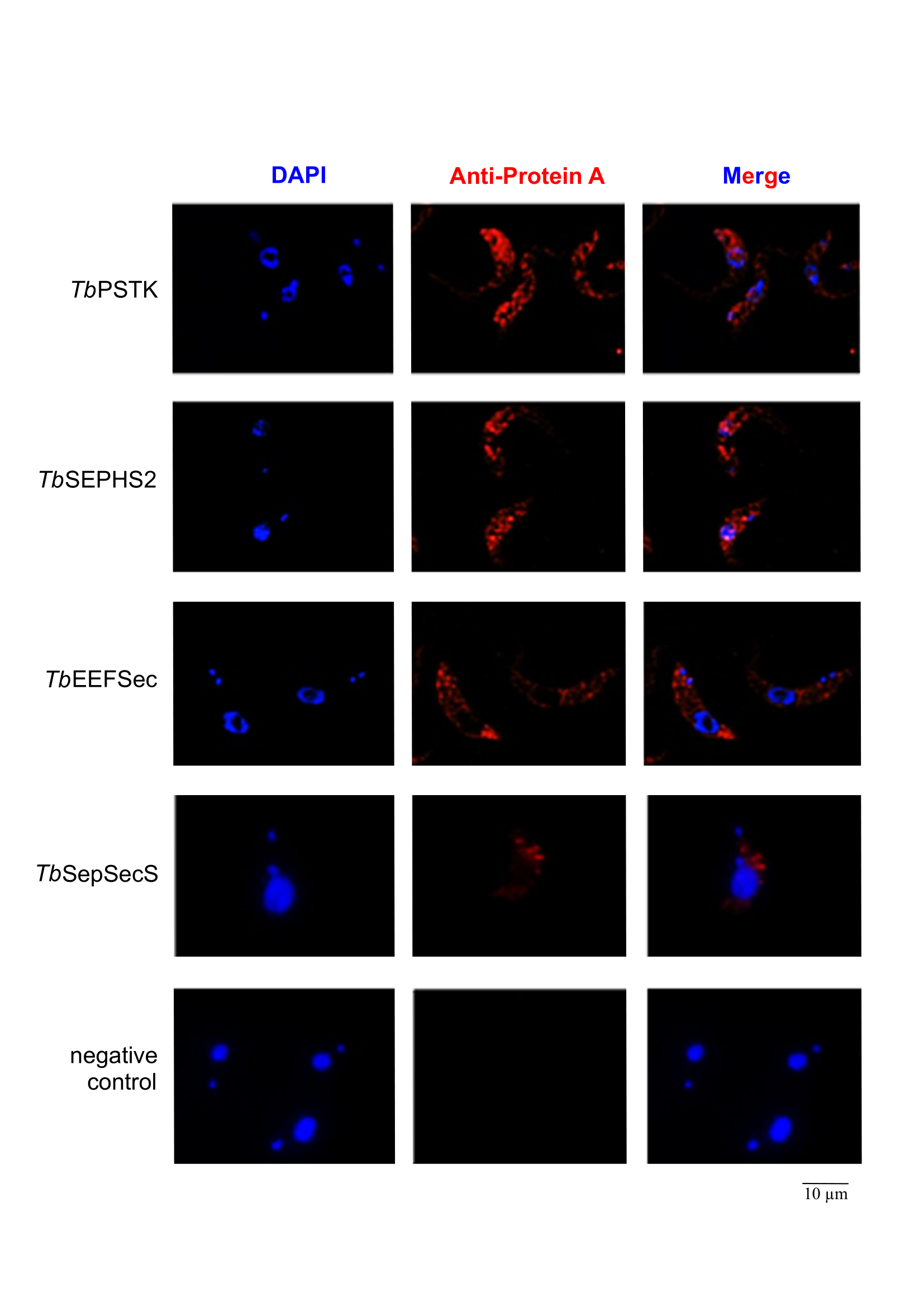

Supplement: S6 Fig — PTP-tagged proteins immunolocalized using anti-protein A antibody (red). DAPI (blue) is used as a nuclear/kinetoplast marker. Untransfected procyclic T. brucei 427 cells were used as negative controls. (TIF) [file pntd.0008091.s006.tif]
