# Supplementary material for: Trypanosomatid selenophosphate synthetase structure, function and interaction with selenocysteine lyase
Source: PLoS Negl Trop Dis. 2020 Oct 5;14(10):e0008091. doi: 10.1371/journal.pntd.0008091 (PMC7595633; doi:10.1371/journal.pntd.0008091)
Supplement: Attachment — Submitted filename: renamed_cca5f.docx [file pntd.0008091.s009.docx]

In my opinion, elucidating the synthesis of selenoproteins is challenging mainly because this pathway seems to be indispensable for *Trypanosoma brucei* and closely related protists. In this work, Túlio Alves da Silva et al. provided a detailed structural biochemical and functional analysis of trypanosomatid selenocysteine synthetase (SEPHS2), a key enzyme in selenoproteins biosynthesis. The crystal structure of SEPHS2 revealed conserved features important for its function. Next, close interaction between SEPHS2 and selenocysteine lyase forming a binary complex was demonstrated. However, the formation of a higher complex with the other members of the pathway was not proven. The silencing of SEPSH2 by RNAi does not result in a growth phenotype. Only when ER stressors such as DTT and tunicamycin were added, growth defect was observed. Also, the downregulation of selenoprotein T, which is found in the ER of other organisms, resulted in growth defects only in the presence of these chemicals. These findings suggest a possible role for *T. brucei* selenophosphate synthetase in the regulation of the ER stress response.

In conclusion, my opinion on this manuscript is quite positive, and the manuscript meets all the criteria for publishing in PLOS Neglected Tropical Diseases. However, I have several points that need to be addressed. The main criticism lies in the mislabelling of the figures, their numbering, or their citations in the manuscript.

Major points:

1. Graphical scheme for the synthesis of selnocysteine with respect to the localization of individual proteins would significantly improve the manuscript and help the reader with orientation in the topic.
2. The text of the manuscript does not refer to Figure 1D and 1F. On the other hand, the text refers to Figure 1H, which is not present in the panel of Figure 1.
3. The sentence: “Additionally, no stable protein complex was observed for TbEFSec-PTP” refers to Figure 3E, but correct is Figure 3D.
4. The sentence: “RT-PCR analysis revealed that tRNA[Ser]Sec co-precipitates with the TbPSTK-PSEPSECS complex and with TbeEFSec.” refers to Figure 3D, but the correct is Figure 3E.
5. Figure 3: Panel of Figure 3F is not labeled
6. Figure S6: Please provide the figure with proper resolution; the current version is pixelated.
7. As you also mentioned in the text, the detection of the TbSEPSH2 using the C-terminal PTP tag may interfere with the localization. Just to let you know, there is a clear nuclear localization of TbSEPSH2 with the N-terminal *in situ* tag in TrypTag database.
8. Since one of the main conclusions of the paper is the role of selenocysteine biosynthesis in the regulation of ER stress. Do you have any evidence of the localization of *T. brucei* selenoprotein T into ER?
9. Can you discuss in more detail the role of selenoprotein T in the regulation of the ER stress?
