# Supplementary material for: Trypanosomatid selenophosphate synthetase structure, function and interaction with selenocysteine lyase
Source: PLoS Negl Trop Dis. 2020 Oct 5;14(10):e0008091. doi: 10.1371/journal.pntd.0008091 (PMC7595633; doi:10.1371/journal.pntd.0008091)
Supplement: Attachment — Submitted filename: PNTD_D_19_02165_Response.docx [file pntd.0008091.s010.docx]

São Carlos, SP, 28^th^ of June, 2020

Dr. Igor C. Almeida
Associate Editor
PLOS Neglected Tropical Diseases

Dear Dr. Dr. Igor C. Almeida,

Find bellow our point-by-point response and actions taken regarding the reviewer's comments and suggestions to the manuscript entitled “*Trypanosomatid selenophosphate synthetase structure, function and interaction with selenocysteine lyase*”. We reproduced each section of the reviewer's comment, consecutively, followed by our reply or the description of the action taken. The different sections of the reviewing form were reproduved, in bold.

We have addressed and justified all the comments in this revised version of the manuscript as well as we clarified all the reviewer´s concerns. We thank the reviewers for the careful evaluation, comments, and suggestions that improved the present paper.

Sincerely,

Otavio Henrique Thiemann

Structural Biology Laboratory

Physics Institute of São Carlos

University of São Paulo – USP

Av. Trabalhador Sãocarlense 400	Phone:	(55-16) 3373-8089

13566-590 - São Carlos – SP	Fax:	(55-16) 3373-9881

Brazil	E-mail:	thiemann@ifsc.usp.br

**Reviewer #1:**

The manuscript by Thiemann and coworkers provides a lot of new information about proteins involved in selenocysteine biosynthesis in Leishmania and Trypanosoma, especially about the selenophosphate synthetase of these parasitic protists. The work builds upon previous research by this group. The information is novel and interesting. The research has been well performed and the results are discussed in great detail and placed in context. The manuscript has been well written. Nonetheless, I have a number of comments, questions and suggestions concerning the scientific content, and identified a number of minor issues about the presentation.

**Reply-Action:** We would like to thank Dr. Paul Michels for reviewing our manuscript. His comments, questions and suggestions were addressed in the following sections.

**Reviewer #2:**

The study by da Silva et al. addresses structural, biochemical and biological aspects of several components of the selenocysteine metabolism of African trypanosomes. Following the sucessfull crystallization of the selenophosphate synthase, they solve and describe the 3D structure of an N-terminally truncated for of this protein. They investigated the quaternary arrangement of the protein by means of ultracentrifugation and gel filtration approaches, and showed in vitro and in cell the formation of an heterocomplex between the selenophosphate synthase, with selenocystein synthase. Using heterologous-complementation assays, they also demonstrate the residues and regions functionally relevant for selenophosphate synthase activity. Finally, they addressed a potential role of the selenophosphate synthase and the selenocysteine-containing protein SELENOT in the response to reductive stress.

Overall, the methodological strategy and results are consistent with most of the conclusions elaborated.

**Reply-Action:** We would like to thank Reviewer #2 for reviewing our manuscript. The reviewer’s comments, questions and suggestions were addressed in the following sections.

At some point the authors should make clear why this metabolism deserves interest, taking into account that it has been shown not to be indispensable for the in vitro and in vivo survival of the clinically relevant stage of African trypanosomes (as well as for the insect stage). Is there any possibility that this metabolism may play a role during parasite differentiation?

**Reply-Action:** The selenocysteine biosynthesis pathway has been shown by other groups to be dispensable for T. brucei under laboratory controlled conditions. We stated in the synopsis that “This has intrigued us, and lead to question that if maintained in the cell it should be under selective pressure and therefore be necessary.” Our group previously showed that TbSEPHS2 has a potential role in oxidative stress both in PCF and BSF T. brucei. Here, we complemented this information showing a growth defect in PCF and BSF T. brucei under reductive/ER stress upon TbSEPSH2 ablation.

To address the reviewer request, we added to the Discussion section the following: “The ER function is particularly necessary for efficient production of GPI-anchored procyclins that coat PCF T. brucei and of variant surface glycoprotein (VSG) proteins [32] that protects BSF T. brucei surface from effectors of the host immune system [67]. However, little is known about the exact role of the ER in T. brucei developmental differentiation”. Among the selenoproteins that have been shown to be ER residents in mammals, SELENOT and SELENOK are also conserved in T. brucei. We added to the Discussion section: “The TrypTAG database contains subcellular localization data for a GFP-tagged TbSELENOT as reticulated in the cytoplasm of the procyclic T. brucei, which is consistent with an ER localization. It will be interesting to evaluate its co-localization with ER markers.”

We also added to the Discussion: “Apart from SELENOT, mammalian SELENOK has been associated with ER homeostasis by promoting calcium flux. However, we were unable to generate a stable SELENOK-RNAi T. brucei cell line. It will be informative to evaluate its function in T. brucei since our results from ER stress in selenophosphate synthetase knockdown PCF and BSF T. brucei indicate a putative function for the selenocysteine biosynthesis pathway in T. brucei ER stress. On the other hand, it is also possible that TbSEPHS2 has a more direct role in ER stress protection.”

**Reply-Action:** We stated in the introduction that “Adverse environmental conditions such as nutrient deficiency, hypoxia, oxidative stress, pH and temperature variation occur throughout their life cycle [5]”. We finished the Discussion stating that: “our data stress the need for further investigation of the exact molecular processes involved in T. brucei developmental differentiation and upon oxidoreductive stress, pH and temperature variation in their hosts.”

At several part of the Result and Discussion sections there is redundancy in the presentation and discussion of the results. I would recommend that the authors make an effort to limit themselves to simply describe and interpret the results without major framework discussion.

**Reply-Action:** We revised the entire manuscript to remove the redundant sentences from the text, as indicated by the correction marks. In some sections we believe that some redundancy is necessary for clar

**Key Review Criteria Required for Acceptance**

**Reviewer #1:**

Methodology is generally sound. For minor issues, see section 'Editorial and Data Presentation Modifications'.

**Reply-Action:** All the minor issues pointed out by Dr. Paul Michels were corrected as suggested by him in the following sections.

**Reviewer #2:**

The objectives and methodological design of the study are clear and suited to adress the first. Some concerns are outlined in the sections below.

**Reply-Action:** All the issues pointed out by Reviewer #2 were corrected as specified in the following sections.

**Results**

**Reviewer #1:**

The analysis matches the analysis plan. The results are in general clear and completely presented, and the figures are clear besides some minor issues. For detailed, minor issues, see section 'Editorial and Data Presentation Modifications'.

**Reply-Action:** All the issues pointed out by Dr. Paul Michels were corrected as suggested by him in the following sections.

**Reviewer #2:**

The results match the analysis plan however some results are not properly presented (see comments below). Figures and images are well presented (see minor comments in Section Data Presentation Modifications).

**Reply-Action:** All the issues pointed out by Reviewer #2 were corrected as suggested by him in the following sections.

**Additional comments:**

-  the truncated form of SEPHS2 was cryztallized as a monomer, however, in solution, the protein appears to display a dimeric arrangement. May the author comment whether the N-terminal portion of SEPHS2 contributes to protein dimerization?, another question, is wether the disordered N-terminal region of SEPHS2 may contribute to an anomalous behavior of the protein in solution; May the autors add to the Table from Fig. 1G the theoretical sedimentation coefficient expected for a SEPHS2 monomer?

**Reply-Action:** The disordered N-terminus of the protein does not seem to contribute to an anomalous behaviour of the protein in solution. In fact, the CD spectra (Figure S4A) of the truncated forms indicate that there is no major alteration in the secondary structure of the protein upon truncation. Also, native gel electrophoresis (Figure S2) indicates that the truncated proteins still dimerize in vitro. Finally, the truncated proteins exhibit residual ATPase activity (Figure 2A) indicating that ATP sites can still form in the absence of the N-terminal region of the protein. Furthermore, we were able to apply the crystallographic symmetry to identify the likely dimerization interface of truncated *L. major* SPSH2 although only its monomer was found in the asymmetric unit of the crystal. In order to clarify this concern in the text, we added the following statement: “Furthermore, the disordered N-terminus of the protein does not seem to be necessary for *L. major* and *T. brucei* SPSH2 dimerization and its absence does not seem to destabilize their secondary structures.”

The theoretical sedimentation coefficients (ssphere) calculated using SEDNTERP for the dimer and the tetramer oligomers of T. brucei and L. major SPSH2 were added to Figure 1F. This value corresponds to the sedimentation coefficient of a sphere with the molecular weight and density of the corresponding protein. As we can see in Figures 1D and 1F, the measured sedimentation coefficient is larger than s­sphere for the dimers, indicating that they are elongated molecules which corroborates the structural model for the dimer. This information was added to the figure legend.

- please indicate in the legend of Fig 3C the concentration of each protein species used for the titration as well as the corresponding mass centers of the spectrum for each condition tested protein

**Reply-Action:** The information about protein concentration was added to Figure 3 legend and the corresponding mass centres of the SEC spectra were added to Figure 3A.

-  please indicate whether single clones or whole population of SEPHS2- and SELENOT-RNAi T. bruce were subjected to phenotypic analysis.

**Reply-Action:** As stated in the Methods section, “The RNAi constructs were obtained according to Costa et al. 2011. Transfected cells were cloned by limiting dilutions and selected with 2.5 μg/mL of phleomycin (Sigma)”. We clarified this information in the text as follows: “The RNAi constructs were obtained according to Costa et al. 2011. Clonal populations of transfected cells were obtained by limiting dilutions and selected with 2.5 μg/mL of phleomycin (Sigma)”.

- please check the following text because the references to Figs appears to be missquoted "Interestingly, SELENOT-RNAi-induced PCF cells were more sensitive to TN than DTT (Figures 6F and 6H). On the other hand, sensitivity to different concentrations of DTT varied in SELENOT-RNAi- BSF T. brucei, with induced cells being more sensitive to 350-400 uM DTT. No significant effect was observed in the presence of TN (Figure 6E-H)." BSF T. brucei depleted in SELENOT did not show any significant sensitivity towards DTT or TN, when compared to non-induced parasites.

**Reply-Action:** We replaced this sentence with the following: “Interestingly, SELENOT-RNAi-induced PCF cells were only sensitive to intermediate concentrations of DTT (1.0 nM) and TN (10 µM) (Figures 6E and 6G, repsectively). On the other hand, no significant effect was observed for SELENOT-RNAi- BSF T. brucei in the presence of DTT or TN. (Figures 6F and 6H, respectively).”

- for the following sentence: "Treatment with different concentrations of hydrogen peroxide did not affect T. brucei growth (Figure 6J-K),", please highlight that such conclusion is valid for both parasite life stages: PCF and BSF.

**Reply-Action:** We clarified this statement in the text as follows: “Treatment with different concentrations of hydrogen peroxide did not affect the growth of any of the SELENOT-RNAi T. brucei forms upon SELENOT depletion (Figures 6K and 6L).”

**Conclusions**

**Reviewer #1:**

The conclusions drawn from the data are in general warranted.  The Discussion is detailed, appropriate and places the result in the biological context. For detailed, minor issues, see section 'Editorial and Data Presentation Modifications'.

**Reply-Action:** All the issues pointed out by Dr. Paul Michels were corrected as suggested by him in the following sections.

**Reviewer #2:**

Most conclusions are correct, except for some related to the biological results obtained with different RNAi cell lines. For instance, some conclusions related to the sensitivity of the RNAi cell lines towards DTT or tunicamycin are somehow (and likely unvoluntary) missleading.
The limitations of the present study and the prospective for new assays demanded to solve some questions are well identified by the authors.
For instance, in the Synopsis and in the Conclusions it is stressed the concept that "Our results also show how the interaction of different proteins leads to the protection of the cell against the toxic effects of seleium compounds during selenocysteine synthesis". I disagree wit this statement since depletion of SEPHS, which should lead to an accumulation of toxic selenium, proved not to entail any detrimental effect on procyclic and bloodstream T. brucei (Ref. 11 and 27). Even for both parasite stages of T. brucei, the KO of SEPSECS, which should lead to an accumulation of toxic selenium metabolites, did not render a defective phenotype in vitro and in vivo (Ref. 11, 27, 28). Instead, may it be possible that the SEPHS2/SCLY complex warrants an efficient recycling of the trace element Selenium?

**Reply-Action:** We replaced the statement in the synopsis with the following sentence: “Also, extensive and dynamic protein-protein interactions must happen to deliver selenium-containing intermediates along the pathway in order to warrant efficient usage of biological selenium in the cell”. We also replaced the statement in the Discussion with the following sentence: “Together, our data suggest that both trypanosomatid selenoprotein biosynthesis and recycling are coupled through the SCLY-SEPHS2 direct interaction (Figure 4A), likely warranting the efficient usage of biological selenium in the cell.”

- Please revise the following statement "Here, we demonstrated that ER stress
with DTT and TN upon TbSEPHS2 ablation leads to growth defects in both PCF and BSF T. brucei, indicating a role for TbSEPHS2 in the ER stress response.", because the experimental data presented for BSF and TN do not support such conclusion.

**Reply-Action:** We replaced this statement with: “Here, we demonstrated that ER stress with DTT and TN upon TbSEPHS2 ablation leads to growth defects in PCF T. brucei, while only DTT showed a negative effect in BSF T. brucei cells expressing TbSEPHS2 RNAi. Together, our data indicate a role for TbSEPHS2 in the ER stress response.”

- Please revise the following statement "Indeed, we observed that SELENOT knockdown PCF and BSF T. brucei cells were also sensitive to ER stress using DTT,...". This is not true because data presented in Fig. 6 shows that BSF depleted in SELENOT are fully insensitive to TN and DTT treatment, and PCF showed a marginal sensitivity towards these stressors at single (out of three) concentrations tested. Thus, the behavior was not concentration-dependent and thus, probably irrelevant. If the authors agree with that, then, they should reconsider not to stress, along the text, a role for SELENOT in ER redox homeostasis, as their data are not fully conclusive in this respect.

**Reply-Action:** We have toned down the possible role for SELENOT in ER redox homeostasis. We replaced the statement with: “Among those, SELENOK and SELENOT are conserved in trypanosomatids [12,66]. Interestingly, mammalian SELENOT is localized to the ER and seems to have a thioredoxin-like activity to maintain ER homeostasis [65]. SELENOT knockout led to early rat embryonic lethality and its knockdown in corticotrope cells promoted ER stress and unfolded protein response (UPR) [65]. The TrypTAG database contains subcellular localization data for a GFP-tagged TbSELENOT as reticulated in the cytoplasm of the procyclic T. brucei, which is consistent with an ER localization. It will be interesting to evaluate its co-localization with ER markers. PCF, but not BSF T. brucei were marginally sensitive to DTT and TN at intermediate concentrations. We also verified that both TbSELENOT-RNAi PCF and BSF T. brucei cells were not sensitive to increasing levels of hydrogen peroxide. The mild effect of ER stressors that we observed in SELENOT-RNAi PCF T. brucei and the absence of a negative effect in BSF T. brucei is intriguing.”

- There is not direct evidence from the biological data produced in this study to support the following statement "Our data suggests that ER N-glycosylation of proteins is strictly regulated in trypanosomatids." First, because non of the protein studied in this work was shown to be localized at the ER; second, because the impact on N-glycosylation of downregulating Sel-metabolism in T. brucei has not been addressed in this study.

**Reply-Action:** We removed this sentence from the text.

**Editorial and Data Presentation Modifications?**

**Reviewer #1:**

A general comment: Next time, please number pages and/or lines to make the work of reviewers a bit easier.

**Reply-Action:** We appologyse for the inconvenience, indeed page and line numbering would have facilitated the reviewer´s work.

**Scientific issues:**
1.      Abstract, line 1: The description ‘early-branching eukaryotes’ is outdated since it is known, since about two decades, that they did not branch early from a common eukaryotic phylogenetic trunk as previously thought. The current view is that different superphyla originated already at the beginning of the eukaryotic evolution (LECA) and trypanosomatids belong to a different superphylum (Excavata) than vertebrates/fungi (Opisthokonta).

**Reply-Action:** The issue was corrected in the Abstract.

2.      Abstract, line 17: Should the sentence not state ‘only  SELENOT-RNAi procyclic T. brucei cells were sensitive’ (as opposed to bloodstream cells)?  Nonetheless, I am not convinced by the conclusion, because the effect of DTT between induced and non-induced cells seems only significant for one DTT concentration used, not for the other two (see also related comments below).

**Reply-Action:** We removed this conclusion from the Abstract. We also re-wrote the discussion about the possible role for SELENOT in ER stress response “PCF, but not BSF T. brucei were marginally sensitive to DTT and TN at intermediate concentrations. We also verified that both TbSELENOT-RNAi PCF and BSF T. brucei cells were not sensitive to increasing levels of hydrogen peroxide. The mild effect of ER stressors that we observed in SELENOT-RNAi PCF T. brucei and the absence of a negative effect in BSF T. brucei is intriguing.”

3.      Introduction, end of 4th paragraph: Mention if the TbSEPHS2 RNAi experiments were done with both PCF and BSF cells.

**Reply-Action:** The information was added to the Introduction.

4.      Introduction, 6th paragraph, line13: Same as above: I am not convinced by the conclusion, because the effect of DTT between induced and non-induced cells seems only significant for one DTT concentration used.

**Reply-Action:** We removed this conclusion from the Introduction.

5.      Results, Section 1 (The L. major selenophosphate synthetase ..), end of paragraph 2: What is the basis for the conclusion that ‘Our crystal structure suggests that the conserved AIRS-like fold of selenophosphate synthetases is necessary for their enzymatic mechanism.’? Or did the authors meant to conclude: ‘Our finding that the AIRS-like fold of selenophosphate synthetases is also conserved in our Leishmania crystal structure suggests its necessity for the enzymatic mechanism.’?

**Reply-Action:** We corrected this statement in the Results section.

6.      Results, Section 1, last sentence: I suppose a sulfate ion was identified in the crystal structure.  Could it be highlighted in the cartoon representation (with its mentioning in the legend)?  Moreover, it seems relevant to add the information also in the legend of Fig. S1.

**Reply-Action:** The sulfate ion is shown as sticks (that look like an X – see comment #11) in the representations of the structure. We clarified this information in the Figure 1 and its legend.

7.      Results Section 4 (TbSEPHS2 RNAi-induced …..) and Fig. 5: The observation that low concentrations (0.15-0.30 mM) of DTT affect growth in TbSEPHS2 RNAi-induced BSF cells, but a higher concentration (0.45 mM) not (and all concentrations do in PCF cells) is intriguing and may deserve further discussion.

**Reply-Action:** We revised the discussion to address this issue.

8.      Results Section 5 (Selenoprotein T (SELENOT)): The second part of this section is not easy to follow. I have the impression that, both in the text and the legend, the labelling of panels E – H has been mixed up.  My understanding is that panels E and G are about PCF cells and F and G about BSF cells (= similar organization of panels as in Figure 5).
In that case, the text should probably changed as follows:
line 11: ‘(Figures 6E and 6G)’ to ‘(Figures 6E – 6H)’
line 12: ‘(Figures 6F and 6H)’ to ‘(Figures 6E and 6G)’
line 14: add ‘(Figure 6E-H)’ after ‘DTT’
line 14: ‘(Figure 6E-H)’ to ‘(Figure 6F and 6H)’
and the following change in the legend, line 8:
‘E- and F- PCF T. brucei cells’ to ‘E- and G- PCF T. brucei cells’
‘G- and H- BSF T. brucei cells’ to ‘F- and H- BSF T. brucei cells’

**Reply-Action:** Thank you for pointing out this mistake. We corrected the Figure citation in the text.

Furthermore, I am puzzled by two issues in this section:
(1)     The conclusion mentioned in the Abstract and the end of the Introduction about the sensitivity of RNAi-ablated PCF  cells for DTT (although only significant at one concentration) is surprisingly not addressed in this section.

**Reply-Action:** We corrected the Results section mentioning the results accordingly.

(2)     I don’t see how to conclude from Fig. 6 (panel F?) that ‘On the other hand, sensitivity to different concentrations of DTT varied in SELENOT-RNAi- BSF T. brucei, with induced cells being more sensitive to 350-400 uM’. First, there seem no significant difference between induced and non-induced cells. Second, the figure mentioned 0.60 mM, not 0.40 mM.

**Reply-Action:** We corrected the Results section.

9.      Discussion: The sentence ‘Here, we demonstrated that ER stress with DTT and TN upon TbSEPHS2 ablation leads to growth defects in both PCF and BSF T. brucei, indicating a role for TbSEPHS2 in the ER stress response.’ is not entirely accurate.  As demonstrated in Fig. 5D (and correctly mentioned elsewhere in the manuscript), TN does not have such effect in BSF trypanosomes.

**Reply-Action:** We replaced this statement with: “Here, we demonstrated that ER stress with DTT and TN upon TbSEPHS2 ablation leads to growth defects in PCF T. brucei, while only DTT showed a negative effect in BSF T. brucei cells expressing TbSEPHS2 RNAi.”

10.     Discussion: About the sentence ‘Indeed, we observed that SELENOT knockdown PCF and BSF T. brucei cells were also sensitive to ER stress using DTT, ….’, see my comments above (for PCF cells only significant at one concentration (Fig 6E), for BSF cells results seem not convincing at all (Fig. 6F)).

**Reply-Action:** We corrected this statement as indicated above.

11.     Figure 1: in the various cartoons a loop is highlighted with X. Please explain in the legend.

**Reply-Action:** It corresponds to a representation of the sulfate ion as sticks. It was clarified in Figure 1 and its legend.

**Minor comments on the biological nomenclature used:**

**Reply-Action:** All the minor comments were corrected in the text/Figures as indicated.

1. Synopsis, lines 3 and 6 and Introduction line 1: Replace the old, incorrect word ‘protozoa’ by ‘protists’ (‘parasitic protists’ or ‘protist parasites’).
2. Introduction, line 1 of 5th paragraph: Change old taxonomic name ‘Kinetoplastida’ to the correct ‘Kinetoplastea’.
3. Introduction, 6th paragraph, lines 1 and 2: no upper case E for the (English) word eukaryotes.
4. M&M, RNAi experiments: ‘host strain 29-13’.  29-13 is not a strain but a cell line derived from strain 427.
5. M&M, RNAi experiments: ‘strain 221’.  221 is not a strain but a VAT (Variant Antigen Type expressing VSG no 221) from strain 427.

**Minor comments on the presentation:**

**Reply-Action:** All the minor comments listed bellow were corrected in the text/Figures as indicated in the text.

1. Abstract, line 2: Add s to parasite to give parasites.
2. Abstract, lines 12-13: Sentence not very clear.  Is meant: ‘….. phosphoseryltRNA Sec kinase (PSTK)-Sec-tRNASec synthase (SEPSECS) complex and the tRNASec-specific elongation factor (eEFSec)-ribosome complex’?  If so add ‘complex’ (2x).
3. Abstract line 13: add h to ‘ditiothreitol’ to give ‘dithiothreitol’
4. Introduction, end 2nd paragraph: Change ‘a detailed …. network and … mechanism … remain poorly understood’ to ‘details of ….  remain poorly understood’.
5. Introduction, 5th paragraph, line 12: change ‘result’ to ‘results’.
6. Introduction, 5th paragraph, line 17: add ‘of’ to give ‘decrease of mitochondrial’
7. Introduction, 6th paragraph, line5: Add ‘The to give ‘TbSEPHS2 crystal structure ….’
8. Results, Section 1 (The L. major selenophosphate synthetase ..), line 6: Change A to Å.
9. Results, Section 1, line 27: Fig. 1H is not found.
10. Results, Section 3 (T. brucei SEPHS2 binds …), line 8: add, after ITC: ‘(not shown)’
11. Results, Section 3, line 12: Why ‘Remarkably’ which suggests some surprise?  Would ‘Importantly’, or ‘Noteworthy’ not be better?
12. Results, Section 3,lines 26 and 27:  Are (Figure 3E) in line 26 and (Figure 3D) in line 27 not swapped?
13. Results, Section 3, line 37: Change ‘present mammalian in’ to ‘present in mammalian’
14.  Results, Section 3, line 39: (Figure 4C) should be (Figure 4D), whereas in line 41 (Figure 4D) should be (Figure 4E)
15. Legend Fig 1, line 9: Correct ‘sedmentation’ to ‘sedimentation’
16.  Legend Fig 1, line 11: Change ‘insert’ to ‘inset’. 
17.  Legend Fig 2, line 4: Correct ‘defficient’ to ‘deficient’
18.  Legend Fig 4, line 3: Correct ‘riobosome’ to ‘ribosome’
19.  Legend Fig 6, line 13: Change 0 in H202 to O
20. M&M, at several places: ‘xx mM de HEPES’.  What is de meaning of ‘de’?  (see sections: Recombinant protein purification, Size exclusion chromatography, Fluorescence spectroscopy)
21. M&M, RNAi experiments: SM-9?  Should this not be SM-79 (the medium described for PCF T. brucei by Brun & Schonenberger, Acta Trop, 1979).
22.  M&M, RNAi experiments, line 10: Change ‘it was used the protocol described’ to ‘the protocol used has been described’
23.  M&M, RNAi experiments, line 11: Change ‘sum’ to ‘summary’
24.  M&M, RNAi experiments, line 3 from end: Delete d from ‘induced’
25.  M&M, PTP-tag TAP, line 7: Change ‘digest’ to its plural form
26.  Legend Fig. S1: Mention the indications for secondary structures
27.  Legend Fig. S3, line 4: Change ‘insert’ to ‘inset’

**Reviewer #2:**

Rephrase the following sentences, all of which are confusing:

**Reply-Action:** The sentences were corrected as requested. We thank the corrections that improved the paper.

-  "TbSEPHS2 crystal structure we determined demonstrates that a conserved fold is important for its function."

-Figure 1D is not quoted in the main text. It may removed, or, instead, the B-factors can be shown in the structure shown in Fig1A.

**Reply-Action:** We removed Figure 1D.

- Fig. S5 should be quoted in the following sentence: "Additionally, we observed that ΔN(70)-TbSEPHS2 does not bind TbSCLY as measured by ITC, indicating that the N-terminal region is necessary for in vitro interaction,..."
- In the following sentence, "... for TbeEFSec-PTP (Figure 3E). RT-PCR analysis revealed that tRNA[Ser]Sec co-precipitates with the TbPSTK-PSEPSECS complex and with TbeEFSec (Figure 3D).", swap the quotation to Fig3E and Fig3D, and in the legend of Fig3E, please explain what does input mean.

**Reply-Action:** The sentence was corrected in the text. Input: lysate expressing the respective PTP-tagged protein. IP: Immunoprecipitated complex using anti-IgG beads.

- In teh following sentence "Besides, they are representatives of the Trypanosomatidae family that is evolutionarily distant from the most...", the word "representative" may be replaced by "belong to".
- "SELNOT" should be replaced by "SELENOT" in the following sentence "Interestingly, SELNOT knockout led to early rat embryonic lethality and its knockdown in corticotrope"

**Reviewer #3**

In my opinion, elucidating the synthesis of selenoproteins is challenging mainly because this pathway seems to be indispensable for Trypanosoma brucei and closely related protists. In this work, Túlio Alves da Silva et al. provided a detailed structural biochemical and functional analysis of trypanosomatid selenocysteine synthetase (SEPHS2), a key enzyme in selenoproteins biosynthesis. The crystal structure of SEPHS2 revealed conserved features important for its function. Next, close interaction between SEPHS2 and selenocysteine lyase forming a binary complex was demonstrated. However, the formation of a higher complex with the other members of the pathway was not proven. The silencing of SEPSH2 by RNAi does not result in a growth phenotype. Only when ER stressors such as DTT and tunicamycin were added, growth defect was observed. Also, the downregulation of selenoprotein T, which is found in the ER of other organisms, resulted in growth defects only in the presence of these chemicals. These findings suggest a possible role for T. brucei selenophosphate synthetase in the regulation of the ER stress response.

In conclusion, my opinion on this manuscript is quite positive, and the manuscript meets all the criteria for publishing in PLOS Neglected Tropical Diseases. However, I have several points that need to be addressed. The main criticism lies in the mislabelling of the figures, their numbering, or their citations in the manuscript.

**Reply-Action:** We would like to thank Reviewer #3 for the important remarks and suggestions.

**Major points:**

1. Graphical scheme for the synthesis of selnocysteine with respect to the localization of individual proteins would significantly improve the manuscript and help the reader with orientation in the topic.

**Reply-Action:** We added Figure 4A containing a schematic representation of the current knowledge of the selenoprotein biosynthesis and selenocysteine recycling pathway in trypanosomatids.

2. The text of the manuscript does not refer to Figure 1D and 1F. On the other hand, the text refers to Figure 1H, which is not present in the panel of Figure 1.

**Reply-Action:** We corrected this mistake in text.

3. The sentence: “Additionally, no stable protein complex was observed for TbEFSec-PTP” refers to Figure 3E, but correct is Figure 3D.

**Reply-Action:** We corrected this mistake in text.

4. The sentence: “RT-PCR analysis revealed that tRNA[Ser]Sec co-precipitates with the TbPSTK-PSEPSECS complex and with TbeEFSec.” refers to Figure 3D, but the correct is Figure 3E.

**Reply-Action:** We corrected this mistake in text.

5. Figure 3: Panel of Figure 3F is not labelled

**Reply-Action:** We corrected this mistake in the corresponding figure.

6. Figure S6: Please provide the figure with proper resolution; the current version is pixelated.

**Reply-Action:** We added a high resolution figure.

7. As you also mentioned in the text, the detection of the TbSEPSH2 using the C-terminal PTP tag may interfere with the localization. Just to let you know, there is a clear nuclear localization of TbSEPSH2 with the N-terminal in situ tag in TrypTag database.

**Reply-Action:** We thank the reviewer for the observation.

8. Since one of the main conclusions of the paper is the role of selenocysteine biosynthesis in the regulation of ER stress. Do you have any evidence of the localization of T. brucei selenoprotein T into ER?

**Reply-Action:** We added the following statement to the Discussion: “Interestingly, mammalian SELENOT is localized to the ER and seems to have a thioredoxin-like activity to maintain ER homeostasis [65]. SELENOT knockout led to early rat embryonic lethality and its knockdown in corticotrope cells promoted ER stress and unfolded protein response (UPR) [65]. The TrypTAG database contains subcellular localization data for a GFP-tagged TbSELENOT as reticulated in the cytoplasm of the procyclic T. brucei, which is consistent with an ER localization. It will be interesting to evaluate its co-localization with ER markers.”

9. Can you discuss in more detail the role of selenoprotein T in the regulation of the ER stress?

**Reply-Action:** We added to the Discussion: “Interestingly, mammalian SELENOT is localized to the ER and seems to have a thioredoxin-like activity to maintain ER redox homeostasis [65]. SELENOT knockout led to early rat embryonic lethality and its knockdown in corticotrope cells promoted ER stress and unfolded protein response (UPR) [65].” We also added: “In mammals, SELENOT was identified as necessary for efficient processing of glycosylphosphatidylinositol (GPI) anchoring of proteins [65]. The ER function is particularly necessary for efficient production of GPI-anchored procyclins that coat PCF T. brucei cells and of variant surface glycoprotein (VSG) proteins [32] that protects BSF T. brucei surface from effectors of the host immune system [67]. However, little is known about the exact role of the ER in T. brucei developmental differentiation.” Also, “Apart from SELENOT, mammalian SELENOK has been associated with ER homeostasis by promoting calcium flux.”
